# Fire at Winsley Sanatorium

**Published:** 1913-12-13

**Authors:** 


### FIRE AT WINSLEY SANATORIUM.

Thanks to the pluck and promptitude of some of the staff of Winsley Sanatorium, near Bath, what might have been a very serious fire was prevented in the early hours of last Saturday morning. It appears that about 4.30 a.m. one of the patients saw a red light reflected upon a window, and called one of the nurses. Nurse Hollinwell immediately got up and was quickly joined by three other nurses and the resident medical officer, Dr. C. J. Alexander. The Merryweather portable engine was got out, and while some worked at the pump the others were employed in keeping a continuous supply of water in the tank, and within an hour their efforts were rewarded and the fire was subdued and prevented from reaching the main building. The Sanatorium comprises sixty-eight beds, and has been in process of reorganisation.

